# On the Emergence of Cryptococcus gattii in the Pacific Northwest: Ballast Tanks, Tsunamis, and Black Swans

**DOI:** 10.1128/mBio.02193-19

**Published:** 2019-10-01

**Authors:** David M. Engelthaler, Arturo Casadevall

**Affiliations:** a Translational Genomics Research Institute, Flagstaff, Arizona, USA; b Johns Hopkins University, Baltimore, Maryland, USA; Institut Pasteur

**Keywords:** *Cryptococcus gattii*, Pacific Northwest, black swan, disease ecology, emerging infectious disease, epidemiology, mycology, tsunami

## Abstract

The appearance of Cryptococcus gattii in the North American Pacific Northwest (PNW) in 1999 was an unexpected and is still an unexplained event. Recent phylogenomic analyses strongly suggest that this pathogenic fungus arrived in the PNW approximately 7 to 9 decades ago. In this paper, we theorize that the ancestors of the PNW C. gattii clones arrived in the area by shipborne transport, possibly in contaminated ballast, and established themselves in coastal waters early in the 20th century. In 1964, a tsunami flooded local coastal regions, transporting C. gattii to land. The occurrence of cryptococcosis in animals and humans 3 decades later suggests that adaptation to local environs took time, possibly requiring an increase in virulence and further dispersal. Tsunamis as a mechanism for the seeding of land with pathogenic waterborne microbes may have important implications for our understanding of how infectious diseases emerge in certain regions. This hypothesis suggests experimental work for its validation or refutation.

## Abstract (precis)

The appearance of Cryptococcus gattii in the North American Pacific Northwest (PNW) in 1999 was an unexpected and is still an unexplained event. Recent phylogenomic analyses strongly suggest that this pathogenic fungus arrived in the PNW approximately 7 to 9 decades ago. In this paper, we theorize that the ancestors of the PNW C. gattii clones arrived in the area by shipborne transport, possibly in contaminated ballast, and established themselves in coastal waters early in the 20th century.

## OPINION/HYPOTHESIS

Starting in 1999 and over the subsequent 2 decades, human and animal cases of Cryptococcus gattii have been identified in the North American Pacific Northwest (PNW) (1). Initial environmental studies found widespread fungal presence within the coastal forests and parks of Vancouver Island, near Victoria, Canada (2). Numerous studies have since found the presence of the three unrelated outbreak clones (known as VGIIa, VGIIb, and VGIIc) in the larger PNW region, including the American states of Washington and Oregon (3–5), with VGIIc largely restricted to the Willamette Valley in Oregon (6). In this essay, we use the nomenclature for the C. gattii species complex (7) and major molecular type (i.e., VG) for consistency with the referenced studies in this report, while being aware that the members of the species complex have been proposed to be designated separate species (8). The PNW disease emergence was unexpected, as C. gattii was thought to be restricted primarily to tropical and subtropical zones, namely, in South America, Africa, Asia, and Australia (9). The hunt to find the cause of the dispersal to this temperate zone soon followed the first human cases, leading to a number of causal hypotheses, including shipment of eucalyptus trees (thought to be a possible dispersal mechanism of C. gattii to parts of central and lower California and Mediterranean countries and even from Australia to South America [10]) or agricultural products (11), ecological niche changes due to global warming (2, 12), ocean and wind currents, movements of animals, and human/mechanical transmission (e.g., contaminated tires, shoes, and crates) (13). Multiple causes may have occurred concurrently or sequentially. Multiple groups (11, 12, 14) have proposed the possibility that C. gattii was dispersed to the region decades prior to the outbreak by some natural or anthropogenic means, which was followed by an unknown niche disturbance that led to subsequent human infections.

**FIG 1:**
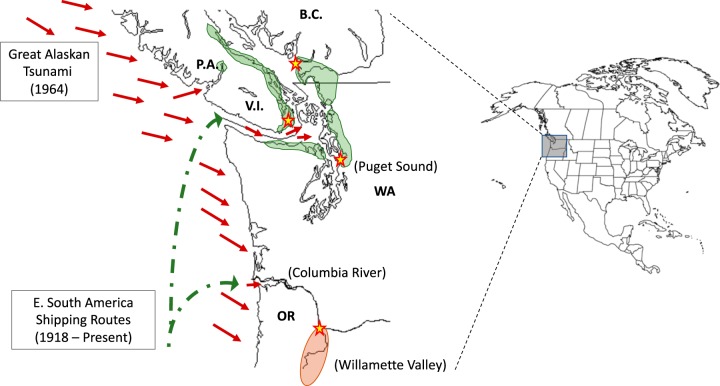
Map of the C. gattii Pacific Northwest dispersal hypothesis. Locales of VGIIa endemicity are shaded green, and the locale of VGIIc endemicity is shaded in orange. Dotted green lines represent PNW maritime shipping routes from eastern South America, and short red arrows represent tsunami wave directions following the 1964 Alaskan Earthquake. Stars represent the capital cities of Portland, OR, Seattle, WA, Vancouver, British Columbia (B.C.), and Victoria, Vancouver Island (V.I.). Also shown is Port Alberni (P.A.) on Victoria Island.

We previously described a hypothetical transport of C. gattii from eastern South American port cities (e.g., Recife, Brazil) to the PNW (including British Columbia, Washington, and Oregon) via contaminated ballast water, due to early shipping between the regions following the 1914 opening of the Panama Canal (15). The timing of this comports with the molecular clock analyses that suggest that the clonal populations of the three C. gattii subtypes identified in the PNW are between 66 and 88 years old (15) (Fig. 1). This would have established populations of C. gattii in the coastal waters of the PNW (where C. gattii has been found and is known to survive for long periods [16]). We now further hypothesize that the PNW coastal forests became contaminated with C. gattii nearly simultaneously from the tsunami waves immediately following the 27 March 1964 earthquake in Prince William Sound, AK.

## 

### The great Alaskan earthquake and tsunamis.

The 1964 Great Alaskan Earthquake was the largest ever recorded in the Northern Hemisphere, registering M9.2 on the Richter scale, second only to the M9.5 1960 earthquake in Chile (NOAA, National Centers for Environmental Information, https://www.ngdc.noaa.gov/nndc/struts/results?bt_0=1964&st_0=1964&type_7=Like&query_7=prince&d=7&t=101650&s=7). The Alaskan Earthquake was felt as far as 4,500 km away, with tidal effects recorded on the Hawaiian Islands. The tidal waves reported in nearby Shoup Bay, AK, were reported to be 67 m high (220 ft), causing significant shoreline devastation. Further to the south, the tsunamis caused significant water surges along western Vancouver Island, most notably in Port Alberni, where dozens of homes were destroyed or washed away. The tsunami continued south, affecting much of the coastline of western North America, even causing several deaths on the beaches of northern California (USGS, Earthquake Hazards Program, https://web.archive.org/web/20141011013757/http://earthquake.usgs.gov/earthquakes/states/events/1964_03_28.php).

It stands to reason (see below) that there has been, and continues to be, a continual presence of C. gattii in the PNW coastal marine environment, again possibly originating from contaminated ballast water from ports from other locales where the organism is endemic. However, the next dispersal leap, the large-scale contamination of the PNW forests, is less obvious. If the coastal marine environment was contaminated first, then there must have been a broad-scale or continual mechanism for aquatic C. gattii to invade the coastal forest of Vancouver Island and other areas of the PNW. If, on the other hand, the ocean-first hypothesis is incorrect and the coastal lands were first contaminated, leading to subsequent coastal and ocean contamination, then we must account for large-scale contamination of the environmental region. Previous hypotheses of original contamination by transport of goods and materials (including plants and trees) require a mechanism for broad dispersal from such material to the larger landscape, including the coastal forests and waters. Ocean environments might be contaminated from snow melt and rain runoff or from contamination from infected sea birds (2), although neither of these phenomena have previously been documented, except for a single report of a C. gattii-positive blue heron (17). It is important to note that, unlike Cryptococcus neoformans, which is classically associated with bird guano, C. gattii, while known to infect multiple bird species, is not shed or otherwise found in guano (18). An alternate hypothesis has been the contact of ocean with contaminated air, as early air sampling studies identified the presence of ambient fungi (likely desiccated yeast cells) in multiple locales on Vancouver Island (2). However, to date, there has been no feasible hypothesis promoted that allows for the initial large-scale land-based contamination of the PNW.

We note that tsunamis have been associated with an increase in fungal diseases (19). Tsunami water can carry pathogenic fungi, as evidenced by case reports of invasive fungal skin and pulmonary disease (“tsunami lung”) in survivors of near-drowning episodes (20–23). Anecdotal evidence for the ability of a tsunami to transport C. gattii comes from a case of cutaneous VGII infection in a survivor of the 2004 Indonesian tsunami in Thailand in which skin injuries presumably became infected by contaminated water (23). These clinical experiences establish that tsunamis can move pathogenic fungi in water flows and provide support for the hypothesis that C. gattii in marine estuaries and other coastal waters in the Pacific Northwest may have reached the land through a tidal wave.

We therefore propose an ocean-first hypothesis, essentially that PNW marine coastlines developed fairly wide-scale marine C. gattii contamination in the decades after one or more initial ballast water-dumping events. Then, on 27 March 1964, the PNW coastal forests became contaminated from the tsunami water surges following the great Alaskan earthquake on that date. This one event, like no other in recent history, caused a massive push of ocean water into the coastal forests of the PNW. Such an event may have caused a simultaneous forest C. gattii exposure up and down the regional coasts, including those of Vancouver Island, BC, Canada, Washington, and Oregon. Local marine populations of select C. gattii strains would have caused local and wide-scale forest contamination events that would subsequently spread more naturally in wooded environments, via other proposed mechanisms both natural (e.g., airborne yeast movements) and anthropomorphic (e.g., fomite-contaminated vehicle tires and shoes) (12, 13, 17). Natural water cycling mechanisms (e.g., snow melt and rain runoff) may account for continued transport of fungi back to the ocean environment, causing cycling of local endemicity. Furthermore, we posit that transport to land in 1964 was followed by a period of soil and tree colonization where C. gattii was exposed to biological and physical selection that possibly increased its infectiousness and virulence for animals, leading to the PNW outbreak 3 decades later.

Multiple pieces of evidence support the ocean-first/tsunami dispersal hypothesis, including (i) evidence of phylogenetic diversification about 50 years ago; (ii) the prevalence of environmental C. gattii predominantly only in coastal forests, rather than further inland, suggesting a connection to the shoreline; (iii) the presence of C. gattii in soils on the Gulf Islands between Vancouver Island and mainland British Columbia; (iv) the presence of C. gattii in humans, mammals, and the forested environment near Port Alberni in central Vancouver Island, an area of the island that was greatly affected by the 1964 tsunami; and (v) the fact that the earliest known case of PNW C. gattii occurred nearly 30 years prior to the 1999 PNW outbreak, establishing a historical record in the region that matches a terrestrial emergence in the 1960s.

**FIG 2:**
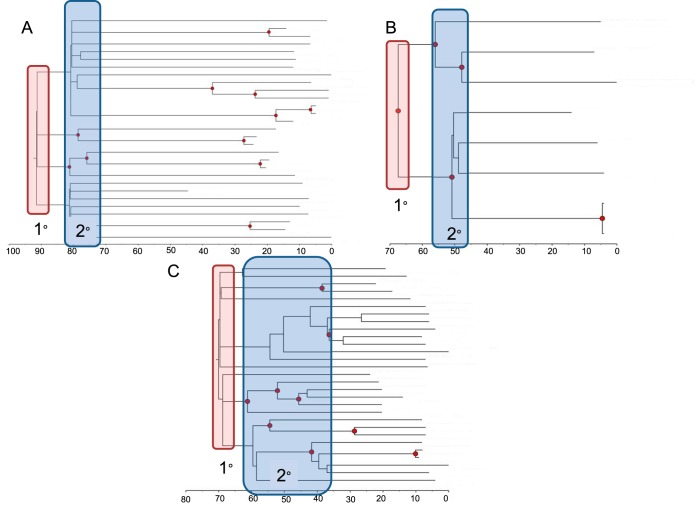
Bayesian phylogenetic analyses of PNW C. gattii samples. The estimated times to the most recent common ancestors (1°; red boxes) were ∼88 years ago (VGIIa) (A), ∼81 years ago (VGIIb) (B), and∼66 years ago (VGIIc) (C). The *x* axis represents years before the present. Blue boxes represent secondary (2°) population divergence events. (Adapted from the work of Roe et al. [15]).

**(i) Phylogenetic evidence.** Phylogenetic analysis of the PNW clones (VGIIa, VGIIb, and VGIIc) provides fairly conclusive evidence of a single introduction event into the PNW followed by local evolution for both VGIIa and VGIIc, whereas VGIIb has a dominant PNW clade resulting from a single primary introduction with possibly one or more additional introductions, with limited evolution and spread (24). The only non-PNW case belonging to the PNW VGIIa clade (besides cases of nonresident travelers to the PNW) was a single isolate obtained from Recife, Brazil, in 1983; all other VGIIa isolates from outside the PNW clade originated from Brazil or elsewhere in the region (24). While not definitively established as originating from Brazil, both VGIIb and VGIIc are most closely related to other VGII lineages from Brazil (and VGIIb has been identified in multiple geographic regions, including Brazil). These data support the “out of Amazon” theory for these subtypes (14, 24). All three PNW clades have been roughly estimated to be 70 to 90 years old using Bayesian statistical inference (Fig. 2) (15), providing for the “Teddy Roosevelt effect” hypothesis, which links the opening of the Panama Canal and subsequent shipping from Brazilian ports to the PNW as a driver of dispersal to the PNW region (15). While specific exported materials that may have been contaminated with C. gattii have not been identified, shipping most certainly would have carried ballast water (and likely microbial contaminants) between the regions. While such molecular clock analyses can provide only relative dating accuracy, closer analysis of the phylogenetic trees from these studies suggests that secondary diversification events, which define much of the current population structure, appear to have initiated approximately 1 to 2 decades following the introduction of each of the clones (Fig. 1). A 1964 tsunami (54 years prior to the above-described analyses) may account for the timing of these population diversification events. Limited tertiary structure suggests limited subsequent evolution beyond these secondary events; therefore, some phenomenon (e.g., simultaneous seeding of the terrestrial landscape) seems to have established multiple individual sublineages that have had restricted subsequent diversification.

**(ii) Environmental evidence.** *(a) Trees and soils.* The early environmental analyses of VGIIa in British Columbia identified that most C. gattii-positive environmental collections (soils and trees) were in the coastal Douglas fir forests and in coastal western hemlock forests bordering the coastal Douglas fir forest (16) (Fig. 1). While these studies were limited in geographical space, the identified contaminated landscapes did encompass the known locations of human and animal cases. Additionally, further ecological analyses have identified higher levels of soil and tree contamination at low-lying elevations close to sea level (25). This is the expected pattern of a tsunami-caused dispersal from contaminated coastal waters.

*(b) Water.* C. gattii from the PNW has been shown to demonstrate long-term survival (at least 1 year) in ocean water, and immediate environmental studies found multiple positive ocean samples near the Vancouver Island coastline (16). Additionally, dozens of infected cetaceans have been found along the PNW coasts, including on the shores of Vancouver Island and the Gulf Islands (2), since the outset of the 1999 outbreak (1, 26, 27); more recently infected pinnipeds have been documented in the region (28), all suggesting large-scale ongoing contamination of the marine environment. Numerous *Cryptococcus* species are adapted to and live in marine water (29–32). Interestingly, C. gattii (and C. neoformans) has an intrinsic mechanism to support cell buoyancy; specifically, its ability to increase capsule production decreases cell density and supports buoyancy in marine salinity levels (33). Nearly all marine mammal infections have been pulmonary, suggesting infection via inhalation. As marine mammals breathe at the surface of the water, it is possible that C. gattii survives at the sea surface microlayer, as described for other *Cryptococcus* species (34, 35), and is subsequently inhaled during breathing episodes. For this report, we also undertook an initial scan of available metagenomic data sets collected in the PNW area for other purposes and have identified a recent metagenomic study of marine microbial communities from expanding oxygen minimum zones in the Saanich Inlet off southeast Vancouver Island (36), where multiple C. gattii VGII-specific kmer sequences were present in the multiple collected marine sample metagenomes (data not shown). These findings further support the concept of long-term ocean survival of C. gattii in the region. Alternatively, these findings may represent continued seeding of coastal waters by contaminated soil runoff and/or by dispersal of airborne yeast cells from nearby contaminated forests, as suggested previously (2).

**(iii) Geographic evidence.** One of the regions on Vancouver Island to receive the most significant tsunami damage was the town of Port Alberni, tied to an inlet on the west side of the island. Several hours after the Great Alaskan Earthquake struck, multiple waves flowed up the Alberni Inlet, cresting at 8 m and striking the Port Alberni region, washing away 55 homes and damaging nearly 400 others (37). Kidd et al. (16) sampled the Port Alberni region, which is approximately 40 km from the eastern edge of the island, finding C. gattii in the forest near Port Alberni. Multiple environmental samples were found to be positive in that study, and it identified human and terrestrial animal cases in this locale (Fig. 1). Infected sea mammals have also been found in the Alberni Inlet (25). Human and terrestrial and marine animal cases have also been reported along the western coast of Vancouver Island, suggesting that coastline contamination has occurred beyond Vancouver’s port region and that contamination of the Port Alberni region may be due to its coastal contamination (i.e., from the 1964 tsunami event), rather than from terrestrial dispersal from the eastern side of the island.

**(iv) Patient evidence.** One of the confounders for a more recent emergence of C. gattii in the PNW (i.e., <50 years ago) is that the earliest known case of C. gattii VGIIa occurred nearly 30 years prior to the 1999 PNW outbreak, a Seattle patient in 1971 (2). Unfortunately, no epidemiologic details exist for the case (e.g., it remains unknown if the case had a history of travel to Vancouver Island); however, this isolate clearly belongs to the PNW VGIIa clade (24), and other cases, human and veterinary, have been subsequently found in the Puget Sound region (38). A scouring of medical records and archived samples has not been able to identify any cases or records of possible C. gattii infection occurring in the region prior to 1970. While only circumstantial, this finding comports with the timeline of environmental seeding from the 1964 tsunami. Additional subsequent infections may have been undetected prior to the 1999 outbreak, especially when viewed in light of the fact that cryptococcal infection can remain dormant after acquisition (39–41).

Much of the above discussion has focused on the dispersal of the VGIIa clone in the Vancouver/Puget Sound region. It is worth noting that the Oregon cases (and all findings of the VGIIc clone) are largely restricted to the Willamette Valley (4, 6, 42, 43), a large river valley south of the Columbia River, which is the primary waterway for shipping to the Pacific Ocean (Fig. 1). The Columbia River inlet of the Pacific is not thought to have been as heavily affected by the 1964 tsunami, with the largest impacts on coastlines occurring nearest the mouth of the inlet (44). Nevertheless, the international shipping port of Portland, further up the Columbia inlet, at the mouth of the Willamette River, may still have acted as a gateway for waterborne C. gattii. The tsunami wave was recorded up to 145 km (90 miles) upriver, including nearly 5-foot waves at the junction of the Columbia and Willamette Rivers (45), which may have transported contaminated estuarine waters upriver. Then a second fluvial event in 1964 consisting of a major flood may have provided another event contributing to the establishment of C. gattii in the area. The 240-km (150-mile)-long Willamette Valley is transected from Eugene to Portland by the Willamette River, which flows north and empties into the Columbia River at Portland Harbor. Nearly 62,000 ha (153,000 acres) of the Willamette Valley was flooded in December 1964, in a devastating “hundred-year flood” (46), causing significant inundation, saturation, and likely contamination of the surrounding region, acting possibly as a similar, yet distinct, water-to-land dispersal mechanism in this southern region of the Pacific Northwest, ironically in the same year as the Alaskan tsunami.

## PATHOGEN DISPERSAL AND BLACK SWANS

The mechanisms of pathogen geographic movement and dispersal are of great interest to ecologists, epidemiologists, medical historians, and others who seek to understand the causes of infectious disease emergences. These mechanisms can be natural or anthropogenic and can include anything from displacement or migration of reservoirs to the landscape, geophysical change, and climate changes to occurrences of natural disasters (as proposed here) (Table 1).

**TABLE 1:** Possible mechanisms of pathogen dispersal to new locales of endemicity

Dispersal mechanism(s)	Natural mechanism(s)	Anthropogenic mechanism(s)
Reservoir movement	Host/vector migration/expansion	Travel, migrations, animal trade
Material movement	Ocean currents	Tires, plants, contaminated materials
Landscape changes	Erosion, flooding	Deforestation, agriculture, cityscapes
Geophysical changes	Continental drift	Major canals and dams
Climate, weather	Ice ages, dust storms	Warming trends
Natural disasters	Earthquakes, tsunamis, floods, tornados, hurricanes	Post-disaster relief efforts (e.g., Haitian cholera)

Many of these are still theoretical mechanisms of dispersal; however, the point is that there are numerous biological, geological, and sociological dynamics on our planet that are likely involved in pathogen evolution and distribution and subsequent disease ecology and epidemiology. A careful study of these dynamics helps us to understand important contributors to the emergence of disease. Our hypothesis of a tsunami-driven contamination of the PNW coastal landscape certainly involves numerous other biological (e.g., the ability of fungi to adapt to coastal waters and coastal climates), geophysical (e.g., the carving of the Panama Canal to allow for easier shipping from eastern South America to western North America), and other anthropogenic (e.g., transport of contaminated materials or ballast between shipping ports) dynamics that would lead to such an unforeseen emergence.

Natural disasters have been well documented to directly and indirectly cause unexpected outbreaks from infectious pathogens, including fungi (19). Famous examples include the remarkable mucormycosis infections caused by deep implantation of *Apophysomyces* during the Joplin, MO, tornado in 2011 (47), the multiple waterborne infections due to exposure to ocean flooding from both the 2004 Indonesian and the 2011 Japanese tsunamis (23, 48, 49) and Hurricane Katrina (50), and increased coccidioidomycosis cases after the 1994 Northridge, CA, earthquake (51). What is less understood and certainly understudied is the effect that these events may have on the actual dispersal of pathogens (and other microbes) into new zones of endemicity, which may result in exposure and disease years to decades later.

Here, we propose that a tsunami was responsible for the large-scale movement of ocean microbes into nearby coastal forests. While the evidence described above is largely circumstantial, the natural disaster dispersal hypothesis should be further explored and tested in other regions and with other microbial populations (see “Hypothesis testing” below). For example, the tsunami hypothesis may also explain how *Cryptococcus* was established in Western Australia following some mechanism of water transport from South America. Such mechanisms of dispersal can be considered “black swans.”

A black swan event, popularized by American financial philosopher Nassim Taleb (52), is an unpredictable event of extreme consequence for which the human tendency is to find oversimplified explanations after the fact. It stems from an ancient thought that all swans must be white, because only white swans had ever been documented until black swans (*Cygnus atratus*) were eventually found in Australia, the discovery of which unraveled previous dogmas. Here, we propose a major new mechanism of pathogen dispersal not previously documented and certainly not predicted, and if true, it will have a disruptive impact on previous dispersal theories, thereby being a prototypical example of a black swan. It is a fact that many major disease events are black swans and therefore typically belie the possibility of prediction. In hindsight, the 2014 outbreak of Ebola in western Africa is thought to have been inevitable, given the combination of considerable human travel and migration between African countries and the pervasiveness of under-resourced health care and public health systems. However, the likely start of the outbreak (i.e., migratory bats falling ill in a hollow tree where children play [53]) was certainly unpredictable, resulting in a high-consequence event and therefore a black swan (54). The continued roulette of influenza virus strain mixing will predictably continue to develop new infectious and possibly pandemic strains in unpredictable black swan ways; e.g., the 2009 swine flu pandemic originating out of Mexico was certainly not predicted. In fact, a review of history shows that most major emerging infectious disease events were black swan events (e.g., the emergence of HIV, severe acute respiratory syndrome/Middle East respiratory syndrome [SARS/MERS], Nipa/Hendra virus, and monkeypox virus in the United States, prenatal effects of Zika virus, and polio-like virus outbreaks from EV-D68, etc.). There is an enormous effort being made with global infectious disease surveillance to identify the earliest time points of emergence of new diseases and outbreaks. These surveillance tools are critical for us to be able to quickly respond and mitigate such events. Significant resources are also being expended for pathogen prediction model development: predicting what, when, and where diseases may emerge prior to detection (e.g., DARPA’s PREEMPT program; https://www.fbo.gov/spg/ODA/DARPA/CMO/DARPA-SN-18-18/listing.html). These efforts are in effect looking for black swans; however, they may fail if such events are truly unpredictable. Taleb (52) argues that, in financial markets, with nearly infinite numbers of seemingly random actions that can act as causes, black swans are truly unpredictable. The response is not to try harder to predict black swans but to work harder to be prepared to respond to them when they do happen, to become “black swan robust.”

The nearly infinite number of small and large stochastic dynamics in the natural and anthropogenic world is probably a good indicator of where we should put our health research dollars when thinking about predicting versus preparing for new emerging infections. In the case of *Cryptococcus* in the PNW, there was a pervasive belief that all C. gattii organisms were found in tropical and subtropical regions; we simply did not predict, nor could we predict, the possibility of the PNW emergence. However, thankfully robust health care, public health, and academic research entities quickly identified and responded to the event. The same was true for HIV, hantavirus, and SARS coronavirus, all on shrinking timescales, due to improvements in technology and communication and advancements in scientific understanding. This cross-disciplinary one-health response between public health, medicine, ecology, and biomedical research is a model that can and should be followed globally to understand and mitigate the effects of the emerging infections that we predict will continue to challenge global public health.

One final note: Western Australia is the region of the continent most prone to tsunamis (55), and it is becoming apparent that at least some populations of Australian C. gattii originated in the western region (i.e., Perth) and have been anthropogenically transported via infected animals (i.e., koalas) to other parts of the continent (56). A tsunami-borne mechanism of transport from ocean to land in Western Australia is highly speculative but certainly possible if the coastal waters were previously contaminated. Ironically, the first actual black swan ever documented was in 1697 in Perth, Western Australia (52), where is it now the official state bird.

### Hypothesis testing.

The hypothesis that C. gattii VGII reached the Pacific Northwest in ship ballast tanks from South America and then became subsequently established on land by a tidal wave suggests several lines of experimentation. Because South American ports are connected to other parts of the world by shipping lanes, other ports must also have become contaminated with C. gattii. The likelihood that C. gattii will become established in a particular locale is probably dependent on the physical and biological conditions operating in that locale. For example, amoebas are important biological control agents for C. neoformans in the environment (57, 58), and the type of microfaunas found in different sites may determine the likelihood for the establishment of an invasive cryptococcal species. Although it is always possible that the marine ecology of the Pacific Northwest provided a unique environment for C. gattii, the more likely scenario is that other coastal areas are also contaminated. Hence, a sampling of water in other ports by deep sequencing may reveal C. gattii-specific genes, thus strengthening the case for shipborne transport. Similarly, sampling of waters in South American ports where rivers flow from the interior of the continent may reveal C. gattii, supporting the notion of fluvial transport by land to sea transport. A more intensive search for new South American isolates may reveal a genetically close relative of the PNW strains, which would support recent transport from that region. Finding C. gattii in other coastal waters without a concomitant increase in cryptococcosis would support the notion that something different happened in the PNW and provide powerful indirect evidence for an unusual event, such as a tsunami. In fact, if C. gattii was found in coastal waters of areas that have suffered tsunamis in recent decades, such as following the Indonesian and Japanese earthquakes of 2004 and 2011, this could allow real-time studies in those areas and perhaps forestall future outbreaks of cryptococcosis in these locales. In this regard, it is noteworthy that the PNW tsunami event was separated from the C. gattii outbreak by 3 decades, suggesting that, if our hypothesis is correct, a period of land adaptation may be required before significant numbers of human and animal cases appear. For example, land adaptation and selection by soil amoeboid predators may have been a necessary condition for increased virulence for mammal infection (59). Evidence for or against a tsunami-related movement from sea to land might come by careful bio-geographic analysis of land areas affected by the tidal wave for the prevalence of C. gattii, with the expectation being that flooded areas harbor more-established sites than nonflooded adjacent lands.
